# Role of golden jackals (*Canis aureus*) as natural reservoirs of *Dirofilaria* spp. in Romania

**DOI:** 10.1186/s13071-016-1524-3

**Published:** 2016-04-28

**Authors:** Angela Monica Ionică, Ioana Adriana Matei, Gianluca D’Amico, Aikaterini Alexandra Daskalaki, Jana Juránková, Dan Traian Ionescu, Andrei Daniel Mihalca, David Modrý, Călin Mircea Gherman

**Affiliations:** Department of Parasitology and Parasitic Diseases, University of Agricultural Sciences and Veterinary Medicine Cluj-Napoca, Calea Mănăştur 3-5, 400372 Cluj-Napoca, Romania; Department of Pathology and Parasitology, University of Veterinary and Pharmaceutical Sciences, Palackého tř. 1/3, 612 42, Brno, Czech Republic; Department of Game and Wildlife, Faculty of Silviculture and Forestry Engineering, Transilvania University, Şirul Beethoven 1, 500123 Braşov, Romania; CEITEC –VFU, University of Veterinary and Pharmaceutical Sciences, Palackého tř. 1/3, 612 42 Brno, Czech Republic; Institute of Parasitology, Biology Centre of Academy of Sciences of the Czech Republic, v.v.i., České Budějovice, Czech Republic

**Keywords:** Wild carnivores, Reservoir, Heartworm, Dirofilariases, Dissemination, Romania

## Abstract

**Background:**

*Dirofilaria immitis* and *Dirofilaria repens* are mosquito-transmitted zoonotic nematodes, causing heartworm disease and skin lesions, respectively, in carnivores. In Europe, the domestic dog is apparently the main definitive host, but patent infections occur also in other species of carnivores. The rapid spread of the golden jackals (*Canis aureus*) throughout Europe opens a question of involvement of this species in the sylvatic cycle of pathogens in the colonised territories, including *Dirofilaria* spp.

**Methods:**

Between January 2014 and May 2015, 54 golden jackals from 18 localities in Romania were examined by full necropsy for the presence of adult filarioid nematodes and blood samples from all animals were screened for the presence of microfilariae of *D. immitis*, *D. repens* and *Acanthocheilonema reconditum* by multiplex PCR DNA amplification.

**Results:**

Nematodes morphologically identified as *D. immitis* were found in 18.52 % of the animals, originating from the southern part of Romania. No *D. repens* or *A. reconditum* were found at necropsy. The molecular prevalence in blood samples from the same animals was 9.26 % for *D. immitis* and 1.85 % for *D. repens*. All samples were negative by PCR for *A. reconditum*.

**Conclusion:**

The relatively high prevalence of *Dirofilaria* spp. infections in golden jackals from Romania together with the increasing density of the jackal populations highlight their potential role in the transmission of these zoonotic parasites and in the maintenance of natural disease foci.

## Background

The main species of *Dirofilaria* infecting European carnivores are *D. immitis,* a widespread nematode that causes severe cardiovascular disease (heartworm disease) [1] and *D. repens*, which localises sucutaneously and is associated with a variety of dermatological clinical signs [2]. Carnivores are the definitive hosts, and the parasites can be transmitted by a large variety of mosquito species (Diptera: Culicidae) [3]. The typical hosts for both species are the domestic dogs (*Canis familiaris*), but patent infections have been reported also in wild canids, such as wolves (*Canis lupus*), red foxes (*Vulpes vulpes)* and golden jackals (*Canis aureus*) [4–6]. Mustelids are likewise involved in the epidemiology of *D. immitis* infection and their reservoir role has been demonstrated in ferrets (*Mustela putorius furo*) [7]. Both *Dirofilaria* spp. are zoonotic; however, *D. repens* is the one involved in most human cases recorded in Europe [8, 9].

Dirofilariases are widely distributed in Europe. The southern countries are historically endemic or even hyperendemic territories, and during the last decades, the infection has spread also into the north-eastern and central Europe [9, 10]. In Romania, infections by *D. immitis*, *D. repens* and *Acanthocheilonema reconditum* have been reported in dogs and humans from different areas of the country [11–13]. However, despite the large populations of wild carnivores present in Romania, the role of these hosts in the spread of filarioid parasites remains unknown.

In the past decades, the populations of golden jackals in Europe underwent considerable expansion. The species originated in the Arabian Peninsula and has initially spread in three directions: westwards to Europe, to Africa and to eastern Asia [14]. More recently, this species underwent further migrations, and its current distribution range covers most of southeastern Europe and parts of eastern and central Europe, with animals occasionally being documented also to the north and the west, far from the established populations [15, 16]. Currently, in Romania golden jackals are largely distributed throughout the country [17].

Golden jackals are known as important reservoir hosts for several parasitic and infectious diseases, many of them having medical or veterinary importance [18, 19]. However, the importance of jackals in the epidemiology of these diseases in newly occupied territories has been poorly investigated. The goal of the present work was to assess the role of the golden jackal as wild reservoir of filarial nematodes in Romania.

## Methods

**Fig. 1:**
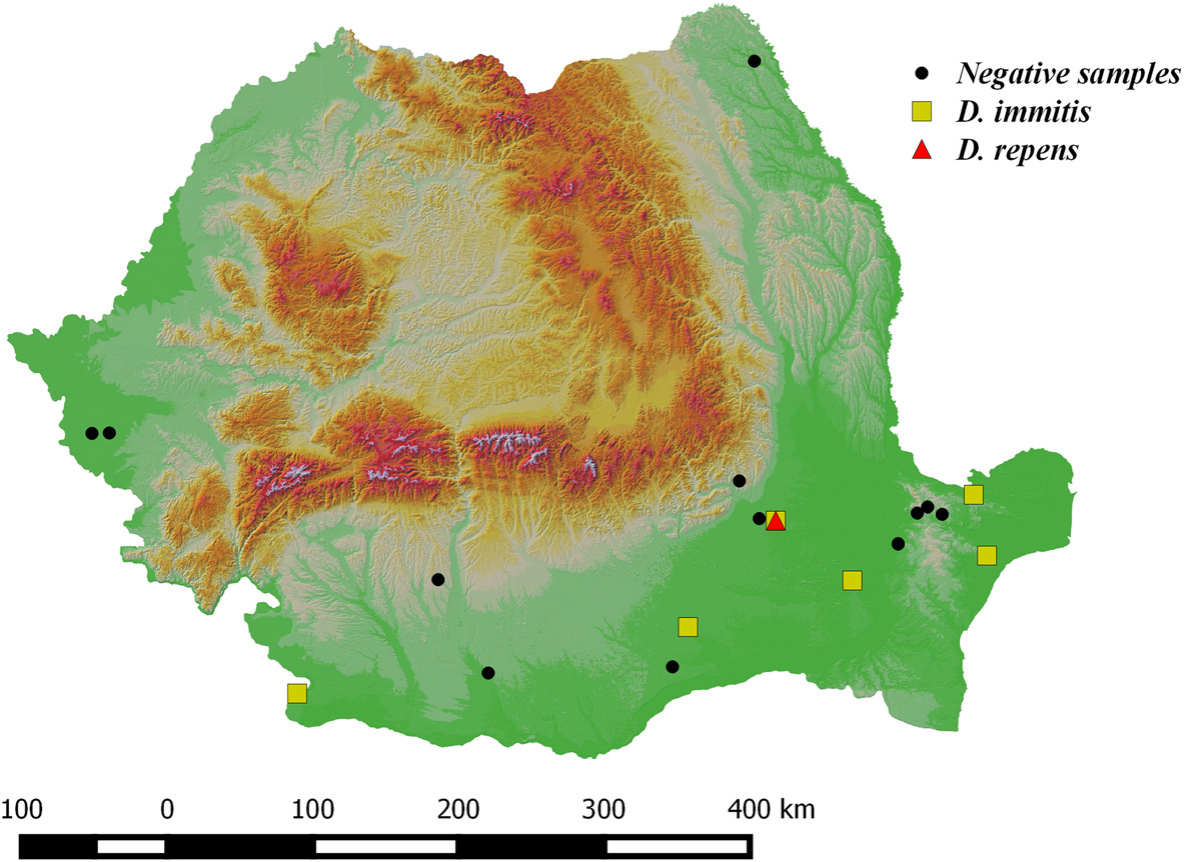
Collection sites and location of positive cases

Between January 2014 and May 2015, 54 legally hunted golden jackals originating from 18 localities of Romania (Fig. 1) were submitted for necropsy at the Department of Parasitology and Parasitic Diseases within the University of Agricultural Sciences and Veterinary Medicine of Cluj-Napoca (Romania). The carcasses were kept frozen at -20 °C until processing. For each animal, the sex, estimated age and location of collection were recorded. Animals were aged based on upper incisive teeth wear [20] and by counting the annual rings of canine teeth cement [21]. The age categories used were juveniles (under 11 months) and adults (over 11 months), according to sexual maturity, which in the golden jackals occurs at the age of 10–11 months [22]. The subcutaneous tissue and muscular fasciae were examined for the presence of *D. repens* and *A. reconditum*. The heart, pulmonary arteries and lungs were longitudinally dissected and macroscopically examined for the evidence of *D. immitis*. All filarial nematodes were collected and identified based on morphological keys and morphometric data available in the literature [23, 24].

Additionally, clotted blood samples were taken from the heart of each examined animal and further processed by molecular methods [25]. Genomic DNA was extracted from 10 individual nematodes (one from each positive jackal) and clotted blood samples using a commercial kit (Isolate II Genomic DNA Kit, Bioline, London, UK) according to the manufacturer’s instructions. PCR amplification reactions were performed as follows: for *D. immitis* a partial cytochrome *c* oxidase subunit 1 gene (*cox*1, ~670 bp) of spirurid nematodes was amplified following [26], while for blood samples a multiplex PCR discriminating three species of filarioids (*D. immitis*, *D. repens* and *A. reconditum*) was performed [27]. PCR products were visualised by electrophoresis in a 2 % agarose gel stained with RedSafe™ 20000x Nucleic Acid Staining Solution (Chembio, St Albans, UK) and their molecular weight was assessed by comparison to a molecular marker (O’GeneRuler™ 100 bp DNA Ladder, Thermo Fisher Scientific Inc., Waltham, MA, USA). PCR products were purified using a commercial kit (QIAquick PCR Purification Kit, QIAGEN, Hilden, Germany) and analysed by sequencing (performed at Macrogen Europe, Amsterdam). The sequences were compared to those available in the GenBank database by using Basic Local Alignment Tool (BLAST) analysis.

Statistical analysis was performed using EpiInfo™ 7 software (CDC, USA). The frequency and prevalence of infection and the 95 % confidence intervals (95 % CI) were established and the differences in prevalence were assessed using chi-square testing. The differences were considered significant if *P*-values were lower than 0.05.

## Results

**Fig. 2:**
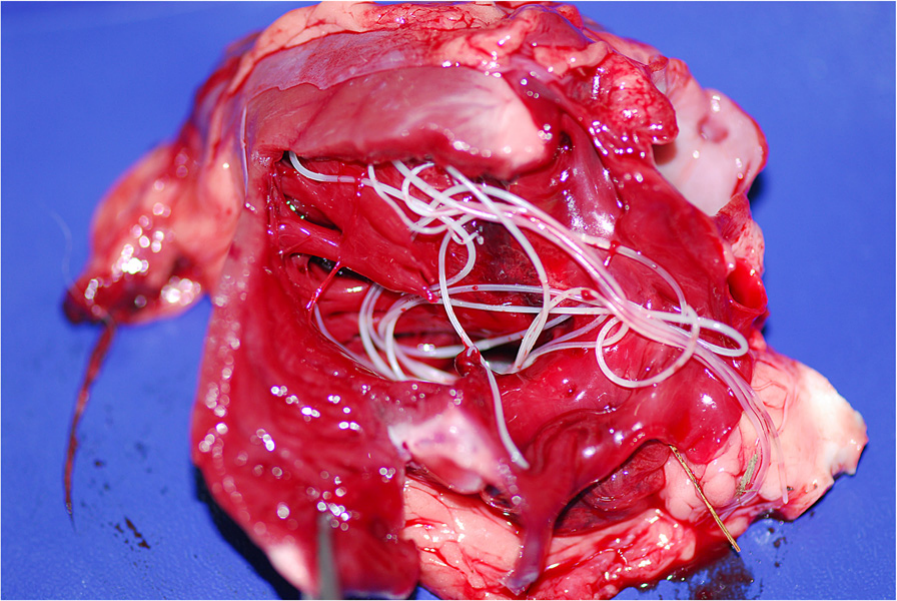
Adult *Dirofilaria immitis* in the right ventricle of a golden jackal

In total, 27 male and 27 female jackals were necropsied; 24 individuals were juveniles, while the other 30 were adults. Nematodes were retrieved from the right ventricle and pulmonary arteries of ten jackals (18.52 %; 95 % CI 9.25–31.43 %) originating from six localities (Figs. 1 and 2). The majority of infected animals were adults (8/10) and the sex ratio was 3:2 in favor of females. The prevalence of infection was significantly higher in adults compared to juveniles (*χ*^*2*^ = 2.90, *df* = 1, *P* = 0.04), whereas the difference between sexes was not significant (*χ*^*2*^ = 0.4909, *df* = 1, *P* = 0.25). The intensity of infection varied between one and seven nematodes/animal (mean intensity three), with seven jackals harboring at least one sexually mature pair (minimum one male and one female).

Overall, 30 nematode specimens were recovered (12 males, 18 females) and identified as *D. immitis* based on morphology and morphometry (Table 1). The identification was further confirmed by randomly sequencing of *cox*1 from ten specimens: one from each positive animal. All sequences were identical. Representative sequence was deposited in the GenBank database (accession number KT716014) and showed a 100 % similarity with strains of *D. immitis* collected from dogs from China and Australia (accession numbers EU159111 and AJ537512).

**Table 1:** Filarioid infection in sampled animals: overall results

Host			*D. immitis*		*D. repens*
No.	Age	Sex	Necropsy	PCR	PCR
1	adult	female	2 males, 5 females	+	–
2	adult	female	2 males, 3 females	+	–
3	adult	male	1 male	–	–
4	juvenile	female	1 male, 1 female	–	+
5	adult	female	2 males	–	–
6	adult	female	1 male, 2 females	+	–
7	adult	male	1 female	–	–
8	adult	male	1 male, 1 female	+	–
9	adult	female	1 male, 3 females	+	–
10	juvenile	male	1 male, 2 females	–	–

Five blood samples were positive by PCR for *D. immitis* (9.26 %; 95 % CI 4.62–15.71 %), one sample was PCR-positive for *D. repens* (1.85 %; 95 % CI 0.05–9.89 %) and all samples were PCR-negative for *A. reconditum*. All five blood samples PCR positive for *D. immitis.* corresponded to animals which were positive also at necropsy (Table 1). We failed to amplify *D. immitis* DNA in blood from five jackals that were found positive at necropsy. In three of these cases, the golden jackals harboured only nematodes of a single sex. In the two cases when both male and female *D. immitis* were present, but with negative PCR results, the animals were juvenile and it is possible that the worms were not sexually mature yet. The prevalence of *D. immitis* infection in adult animals was significantly higher compared to juveniles (*χ*^*2*^ = 2.97, *df* = 1, *P* = 0.04), most likely due to a longer exposure to infected mosquitoes. *Dirofilaria repens* was molecularly identified in one animal (1.86 %) that was also positive for *D. immitis* at necropsy. The *D. repens* sequence was deposited in the GenBank database (accession number KU321603) and showed 95 % similarity to other European strains of *D. repens* (accession numbers KR998257, KR998259, KF692102 and JF461458).

## Discussion

The golden jackal is considered to be one of the most widespread wild canids. In Europe, an ongoing expansion of the Balkan populations has been recorded, their presence having been observed, with records as far west as Switzerland, as far north as Estonia and as far east as the Ukraine and the Russian Federation [15, 16]. In Romania, the first official report of this species dates from 1931, but the first anecdotal historical recording originates from the 18th Century by Dimitrie Cantemir in his work “Descriptio Moldaviae” where the golden jackal is referred to as “*cical*”, an animal coming from the south of the Danube [28]. During the mid-20th Century, the presence of golden jackals was considered occasional, with most individuals originating from the Balkan Peninsula [29]. However, between 1950 and 2000 this species has spread throughout the country, occupying various ecosystems in eastern, southern and north-eastern Romania with specimens being detected in Dobrogea, Muntenia, Oltenia and also in Moldova regions [17, 28]. More recently, golden jackals have also been hunted in western and central Romania [30, 31]. Currently, this species can be found in 28 out of the 41 Romaian counties, with a national population estimated between 6431 and 8923 animals in 2013 [17, 32].

During the last decades, golden jackals have colonised and are currently present in all areas that are endemic for canine dirofilariases in Romania [13, 33–38]. The dissemination of *Dirofilaria* spp. is conditioned by a series of factors, mainly the existence of suitable hosts and the availability of mosquito vectors [39]. Species of mosquitoes belonging to the *Culex pipiens* complex, which are the most efficient natural vectors of *D. immitis* [40], and *Aedes albopictus*, another important natural vector for *Dirofilaria* spp. [41], are commonly found in the study areas [42, 43]. Moreover, in the southern and south-eastern parts of the country, spring and summer temperatures range between 20 and 39.7 °C [44] that are favourable for both, the development of *Dirofilaria* spp. and for a high density of *Culex* spp. [45].

In the present study, the prevalence of *D. immitis* infection established by necropsy was of 18.52 %, showing a relatively high value, compared to other post-mortem studies performed in neighbouring European countries. In Bulgaria, the recorded prevalence ranged between 4.4 % (2/45) and 37.54 % (122/325) [46–48]. In Hungary, 7.4 % (2/27) of examined animals were positive [49]. In Serbia, the prevalence of *D. immitis* in golden jackals was of 7.32 % (32/437) between 2009 and 2013 [50]. Interestingly, PCR reactions revealed a lower prevalence value (9.26 %). This discrepancy can be explained by occult (amicrofilaremic) infections, which may occur under several circumstances, such as the presence of nematodes of a single sex, prepatency of infection or immune-mediated clearance of microfilariae [51]. All individuals that tested negative for *D. immitis* by means of PCR were either harbouring nematodes of the same sex (*n* = 3) or were at juvenile age (*n* = 2), corresponding to the prepatency period, which ranges between six and nine months [52]. Additionally, in the case of mixed infection in jackal no. 4, the presence of *D. repens* together with the immune responsiveness of the host may have inhibited the production of *D. immitis* microfilariae [53].

*Dirofilaria repens* infections have been previously reported in golden jackals from Africa and Asia [4, 54], but this species has never been found in European golden jackals. The presence of this parasite was confirmed solely by molecular means. The adult nematodes were missed during necropsy, probably because of the abundant fat subcutaneous tissue or the extensive bullet wound. The molecular prevalence of *D. repens* was significantly lower (*χ*^*2*^ = 8.1987, *df* = 1, *P* = 0.004) compared to *D. immitis*.

Some authors consider that *D. immitis* infections in golden jackals and wolves are an epi-phenomenon of dog infection, and that they do not represent a real wild reservoir of the infection [3]. Indeed, in wolves *Dirofilaria* spp. infections are only sporadically reported, as individual cases [6, 50, 55–57]. Similarly, several studies suggest that red foxes are not suitable reservoir hosts for *D. immitis* or *D. repens* in some epidemiological settings [25, 49, 58–60]. Indeed, the infection in golden jackals may have originated in infected dogs inhabiting the same areas. However, the high level of patency (9.26 %; 50 % of infected animals) suggests the possibility of subsequent self-sustaining foci within the golden jackal populations. Therefore, golden jackals, due to their increased mobility and spreading, could play a significant role in disseminating *Dirofilaria* spp., particularly *D. immitis*.

Undoubtedly, the domestic dogs are the most abundant carnivores throughout Europe and as such, represent a dominant reservoir of *Dirofilaria* spp. compared to the wild canids in sympatric areas. However, in the present study the overall prevalence of *D. immitis* infection in golden jacakals exceeded that of dogs originating from the same counties (e.g. 25 *vs* 15.94 % in Tulcea County) [13]. The high prevalece of patent *D. immitis* infections in Romanian golden jackals suggests the existence and perpetuation of a sylvatic life-cycle and highlights the importance of jackals as free-ranging resevoirs.

## Conclusion

To the best of our knowledge, the present study reports *D. repens* infection in golden jackals for the first time in Europe. We highlight the relatively high prevalence of patent *Dirofilaria* spp. infection in golden jackals in Romania, underlining their possible role as reservoir host involved in the dissemination of these filarioids, particularly *D. immitis*, in their newly colonised territories.
